# The value of magnetic resonance imaging osteoarthritis knee score in cartilage damage and prognosis of knee osteoarthritis

**DOI:** 10.1097/MD.0000000000044689

**Published:** 2025-09-19

**Authors:** Jinbao Li, Xiuwei Zheng

**Affiliations:** a Radiology Department I, Tianjin Hospital, Tianjin, China.

**Keywords:** cartilage damage, magnetic resonance imaging, osteoarthritis knee score, prognosis

## Abstract

The aim of this study was to investigate the value of magnetic resonance imaging (MRI) osteoarthritis knee score in the assessment of cartilage damage and its prognosis in knee osteoarthritis (KOA). Fifty-two patients with cartilage damage in KOA were selected as the case group, while 50 subjects undergoing health checkups during the same period were selected as the control group. All subjects underwent MRI scans, and patients in the case group received arthroscopy. The severity and prognosis of KOA were assessed by comparing the T2 values of different cartilage regions between the 2 groups using the MRI osteoarthritis knee score. In addition, the preoperative and postoperative visual analogue scale scores of the case group were compared, and the correlation between the T2 values and the MIR osteoarthritis knee score was analyzed. Preoperative T2 values in all cartilage regions of the case group were significantly higher than those of the control group (*P* < .05), with no statistical difference postoperatively compared to the control group (*P* > .05). The preoperative MRI osteoarthritis knee score of the case group was notably higher than that of the control group, and although the postoperative score decreased, it remained higher than the control group (*P* < .05). Visual analogue scale scores significantly improved at all postoperative follow-up points (*P* < .05). T2 values were positively correlated with MRI scores (*P* < .05). MRI osteoarthritis knee score is positively correlated with the T2 value, which may effectively reflect the cartilage damage and prognosis in KOA, with significant clinical application value.

## 1. Introduction

Knee osteoarthritis (KOA) is a common degenerative joint disease, primarily characterized by the degeneration of knee cartilage and bone hyperplasia. As the disease progresses, patients experience symptoms such as knee pain, swelling, and restricted mobility. Cartilage damage is one of the core pathological features of KOA, and its prognosis is influenced by various factors.^[1]^ In recent years, as understanding of the pathological mechanisms of KOA has deepened, researchers have increasingly focused on methods for assessing early cartilage damage and disease progression.

Magnetic resonance imaging (MRI) is a crucial imaging technique used to assess cartilage damage in KOA.MRI can be categorized into 2 major types: morphological imaging and molecular imaging. Morphological imaging is mainly used to assess structural changes in the cartilage, such as cartilage thickness and osteophytes, while molecular imaging focuses on changes in the tissue composition of cartilage, including chondrocytes and extracellular matrix. Molecular imaging can detect changes in sodium ion concentration, proteoglycans, and glycosaminoglycans, thus revealing cartilage damage at the molecular level.^[2,3]^

Histologically, cartilage damage can result from trauma, mechanical load, and other non-physicochemical factors. Cartilage damage may involve various layers, including the superficial, transitional, radial, and calcified layers. Due to the lack of neural, vascular, and lymphatic supply in knee cartilage, its self-repair capacity is severely limited.^[4]^ Moreover, cartilage damage in KOA is not merely a localized alteration; it also induces static changes in adjacent tissues, such as ligament injury and subchondral bone alterations, ultimately exacerbating the patient’s functional impairment.^[5]^

T2-weighted imaging plays a crucial role in assessing changes in the extracellular matrix of cartilage. T2 images reflect changes in extracellular glycosaminoglycans, water content, and proteoglycans, which are vital to cartilage health.^[6,7]^ Different tissue components exhibit varying signal intensities on T2 images, typically represented through pseudocolor mapping.^[8,9]^ These pseudocolor images can assist in detecting changes in cartilage composition, such as the loss of proteoglycans, reduction in water content, and movement of sodium ions, even before significant morphological changes occur.^[10–15]^

Recent studies have shown that T2 values can vary substantially among different cartilage regions due to variations in biomechanical loading, collagen fiber orientation, and local tissue composition. In particular, the lateral femur often demonstrates distinct T2 characteristics compared with the medial femur, likely because KOA-related degeneration more commonly affects the medial compartment, resulting in asymmetric loading patterns. However, the prognostic value of lateral femur T2 changes in KOA remains insufficiently studied, and its relationship with composite MRI scoring systems such as the Whole-Organ Magnetic Resonance Imaging Score (WORMS) has not been clearly established. In the present study, we focused on 5 cartilage regions: the medial femur, lateral femur, medial tibia, lateral tibia, and patellar cartilage. These regions were selected because they represent the major load-bearing and articulating surfaces of the knee joint, where biomechanical stresses and degenerative changes differ substantially. The medial compartment, including the medial femur and medial tibia, is typically subjected to greater mechanical loading and is more frequently involved in KOA pathology. The lateral femur and lateral tibia were included to examine potential inter-compartmental variability and address the limited evidence regarding lateral compartment degeneration. The patellar cartilage was assessed due to its role in patellofemoral osteoarthritis, which can contribute significantly to anterior knee pain. This region-specific approach allows for a more comprehensive evaluation of compartmental pathology and prognostic differences in KOA.

Therefore, the aim of this study was to explore the application value of MRI KOA score in assessing cartilage damage and prognosis of KOA. Through this research, it is hoped to provide new insights and methods for the early diagnosis and progression monitoring of KOA.

## 2. Methods

### 2.1. General data

A total of 52 patients with cartilage damage in KOA treated at our hospital from May 2022 to April 2024 were selected as the case group, while 50 subjects undergoing health checkups during the same period were selected as the control group. In the case group, there were 30 males and 22 females, aged between 38 and 69 years, with a mean age of (56.93 ± 3.64) years. In the control group, there were 29 males and 21 females, aged between 39 and 70 years, with a mean age of (57.01 ± 3.57) years. The baseline data of both groups were comparable (*P* > .05). This study was approved by the ethics committee of Tianjin Hospital. All patients provided written informed consent prior to enrollment in the study.

The sample size was determined based on the total number of patients who met the inclusion criteria during the study period. A retrospective power analysis (α = 0.05, power = 0.8) using the pre–post differences in T2 values as the primary outcome confirmed that the study had sufficient statistical power to detect clinically meaningful differences.

### 2.2. Inclusion and exclusion criteria

Inclusion criteria: patients who met the diagnostic standards for cartilage damage in KOA; patients with complete imaging data; patients who underwent arthroscopic treatment; patients who underwent MRI examination.

Exclusion criteria: patients who did not meet the diagnostic standards; patients with concomitant rheumatoid arthritis, gout, or other rheumatologic immune disorders; patients with tuberculosis, tumors, or other severe systemic diseases; lost to follow-up at any postoperative assessment point.

### 2.3. MRI examination and region selection

All subjects underwent MRI scans using a 3.0T MR system. With the patient in a supine position and the knee joint fully extended, the field of view, layer thickness, and other parameters were set for scanning sagittal 3D fast gradient echo sequence and coronal fat-suppressed fast spin-echo inversion recovery sequence. The T2 values of different regions of the knee cartilage were compared between the 2 groups. T2 mapping imaging data were input into the Syngo workstation, and post-processing software was used to generate T2 mapping pseudocolor images of the knee cartilage. The region of interest was selected, and T2 values of the cartilage were measured directly on the T2 map. Regions of interest were manually delineated in the medial femur, lateral femur, medial tibia, lateral tibia, and patellar cartilage, as these represent the primary load-bearing and articulating surfaces of the knee with distinct biomechanical and pathological relevance in KOA. Each layer was measured 3 times manually, and the average value was taken.

The case group received arthroscopic treatment. The patients were treated with arthroscopic surgery under epidural or general anesthesia. According to the MRI imaging manifestations of KOA, the severity of subchondral lesions was evaluated with reference to the WORMS, with a score of 0 to 5 indicating normal to very cartilage lesions, respectively.

### 2.4. Observation indicators

MRI osteoarthritis knee score was used to evaluate the severity and prognosis of the 2 groups of MRI examinations. The visual analogue scale (VAS) scores of the case group were calculated on the 1st day after surgery, 4 weeks after surgery, and 12 weeks after surgery, and the correlation between T2 values and MRI osteoarthritis knee score was analyzed.

#### 2.4.1. Outcome measures

MRI osteoarthritis knee scores and T2 values of the defined cartilage regions were recorded preoperatively and postoperatively. Pain was assessed using the VAS at baseline, postoperative day 1, 4 weeks, and 12 weeks.

### 2.5. Statistical processing

All analyses were performed using SPSS 26.0 (IBM Corp., Chicago). Continuous variables were expressed as mean ± SD. Pre- and postoperative comparisons within the same patients were analyzed using paired *t*-tests; if the normality assumption (Shapiro–Wilk test) was violated, the Wilcoxon signed-rank test was applied. For variables assessed at multiple postoperative time points (e.g., VAS scores, T2 values), repeated measures ANOVA was used if assumptions of normality and sphericity (Mauchly test) were met; otherwise, the Greenhouse–Geisser correction was applied or the Friedman test was used for nonparametric data, followed by post hoc pairwise comparisons with Bonferroni correction. Pearson or Spearman correlation analysis was used to examine relationships between T2 values and MRI osteoarthritis knee scores, depending on data distribution. Categorical variables were compared using the χ² test. Patients lost to follow-up were excluded from the analysis, and no data imputation was performed. A two-tailed *P* value < .05 was considered statistically significant.

## 3. Results

### 3.1. Comparison of T2 values in different regions of knee cartilage between groups

**Figure 1.**
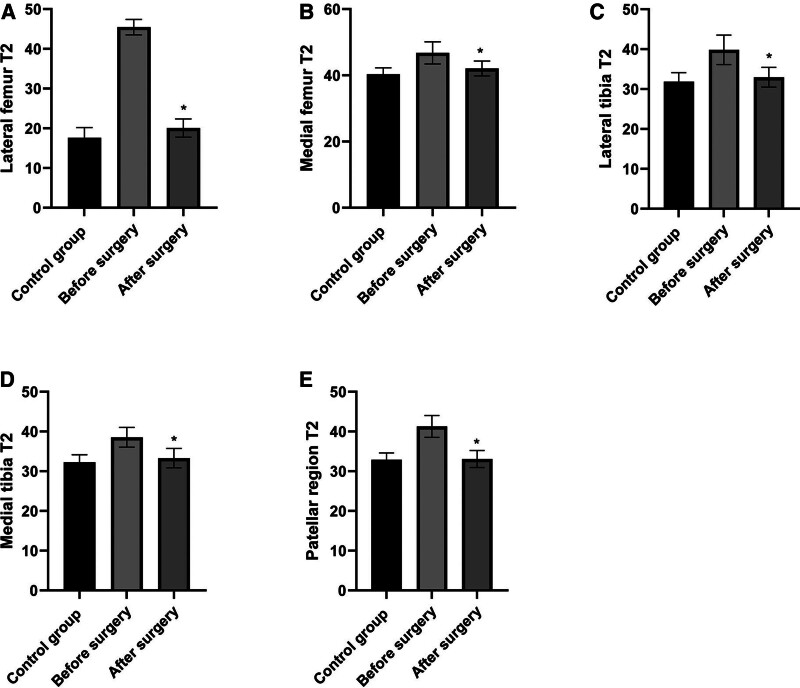
Comparison of T2 values in different regions of knee cartilage between groups. (A) Lateral femur. (B) Medial femur. (C) Lateral tibia. (D) Medial tibia. (E) Patellar region. Note: Compared with before surgery, ^*^P < .05.

The cartilage T2 values of the medial femur (46.76 ± 3.35) ms, lateral femur (45.47 ± 1.93) ms, medial tibia (38.53 ± 2.47) ms, lateral tibia (39.83 ± 3.68) ms, and patellar region (41.28 ± 2.75) ms in the case group were significantly higher than those in the control group before surgery (*P* < .05). After surgery, the cartilage T2 values of medial femur (42.08 ± 2.24) ms, lateral femur (20.06 ± 2.31) ms, medial tibia (33.29 ± 2.42) ms, lateral tibia (32.95 ± 2.47) ms, and patellar region (33.08 ± 2.16) ms in the case group were not statistically different from those in the control group (*P* > .05; Fig. 1, Table 1).

**Table 1:** Comparison of T2 values in different regions of knee cartilage between groups (Mean ± SD, ms).

Group	Lateral femur	Medial femur	Lateral tibia	Medial tibia	Patellar region
Case group (n = 52)					
Before surgery	45.47 ± 1.93*	46.76 ± 3.35*	39.83 ± 3.68*	38.53 ± 2.47*	41.28 ± 2.75*
After surgery	20.06 ± 2.31	42.08 ± 2.24	32.95 ± 2.47	33.29 ± 2.42	33.08 ± 2.16
Control group (n = 50)	17.62 ± 2.57	40.32 ± 1.96	31.87 ± 2.25	32.29 ± 1.86	32.89 ± 1.72
95% CI of difference	26.97–28.73	5.38–7.50	6.78–9.14	5.39–7.09	7.50–9.28
Cohen d	12.29	2.34	2.60	2.85	3.64
*F*	22.054	11.273	14.486	15.019	17.268
*P*	.000	<.001	.000	.000	.000

Compared with before surgery.

* *P* < .05.

### 3.2. Comparison of MRI osteoarthritis knee scores between groups

**Figure 2.**
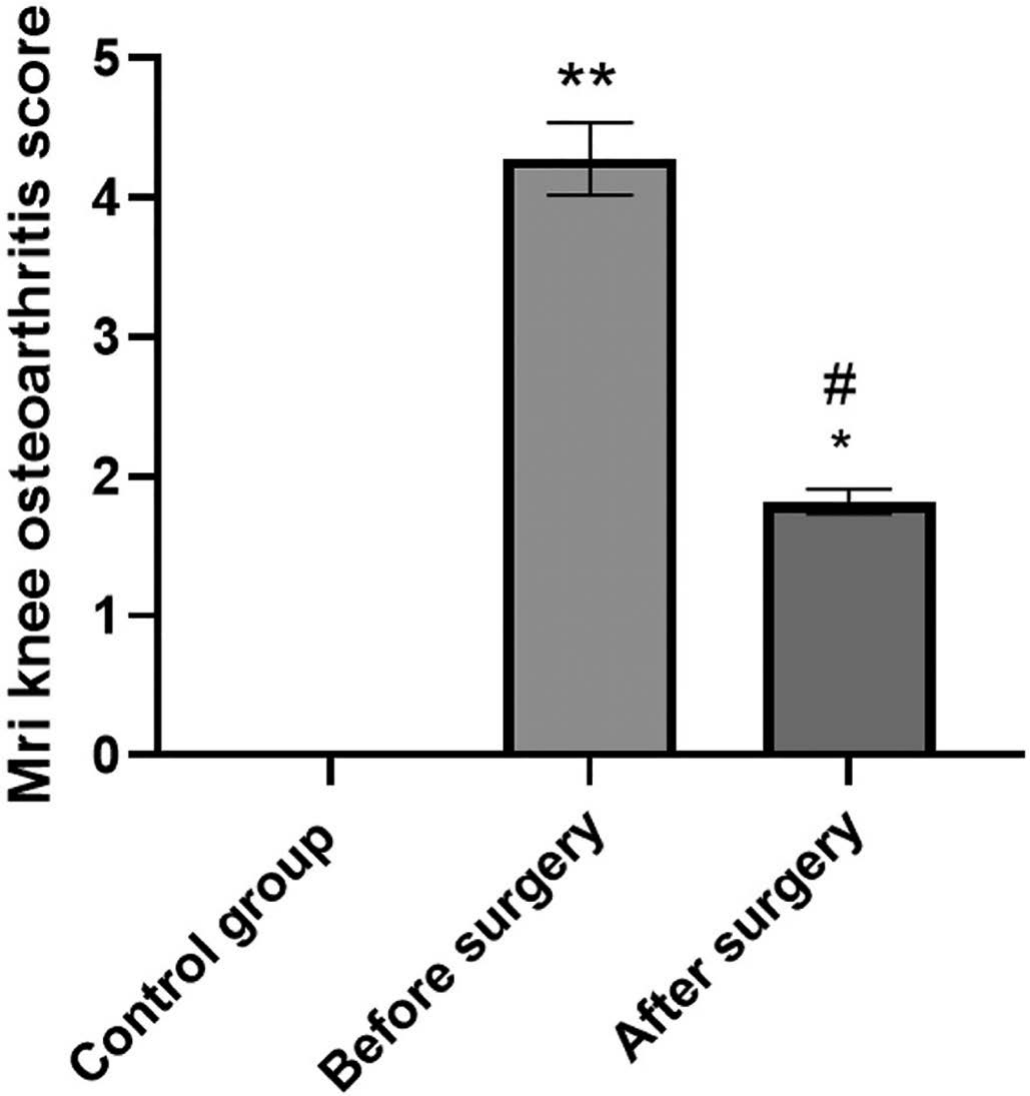
Comparison of MRI osteoarthritis knee scores between groups. Note: compared with the control group, ^*^P < .05, ^**^P < .01; compared with before surgery, ^#^*P* < .05. MRI = magnetic resonance imaging.

The MRI osteoarthritis knee score of the case group before surgery (4.28 ± 0.26) was elevated compared with that of the control group (0) and after surgery (1.82 ± 0.09), and the MRI osteoarthritis knee score was elevated after surgery compared with that of the control group, exhibiting statistically significant difference (*P* < .05; Fig. 2).This residual postoperative elevation suggests the presence of persistent structural abnormalities detectable by MRI, which may represent irreversible cartilage changes despite marked clinical improvement. Such findings could have prognostic implications, indicating potential vulnerability to future degeneration even after successful symptom relief.

### 3.3. Comparison of preoperative and postoperative VAS scores in the case group

**Figure 3.**
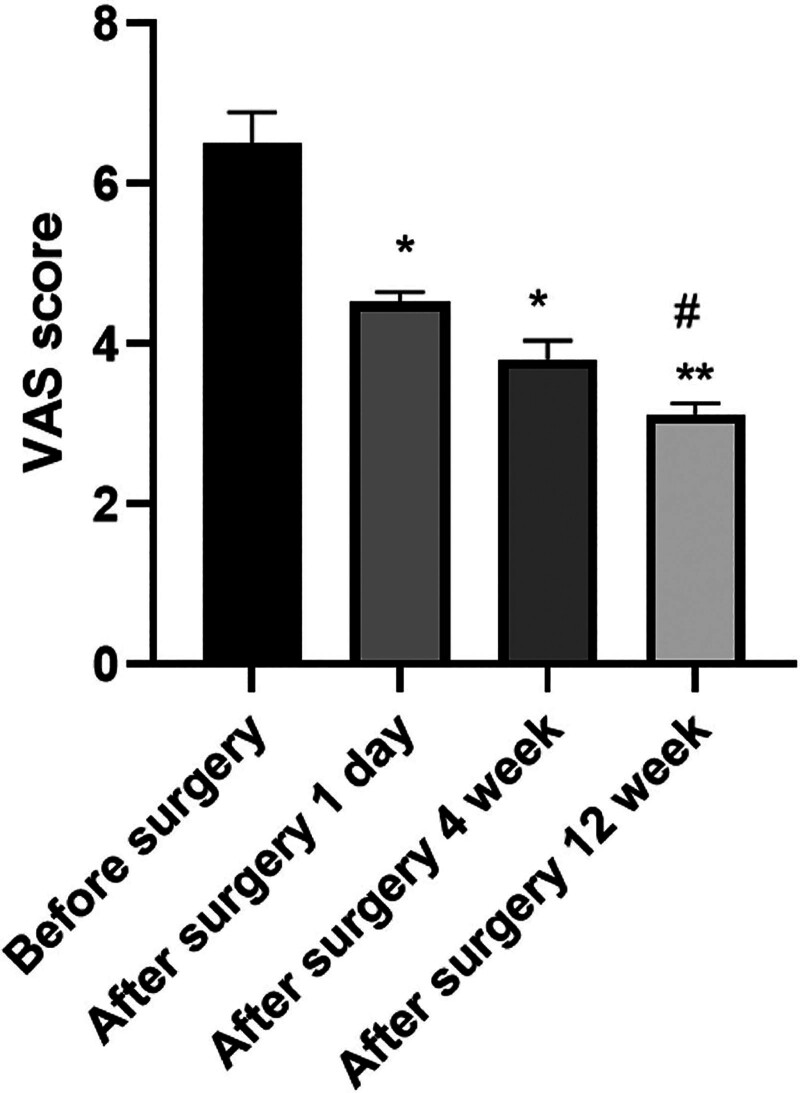
Comparison of preoperative and postoperative VAS scores in the case group. Note: compared with before surgery, ^*^P < .05, ^**^P < .01; compared with 1 day after surgery, ^#^*P* < .05. VAS = visual analogue scale.

The differences in VAS scores of the case groups were statistically significant when compared the preoperative and postoperative follow-up time points (*P* < .05). Compared with before surgery, VAS scores decreased in both groups on the 1st day, 4 weeks, and 12 weeks after surgery, and the decrease was more significant at 12 weeks, showing statistically significant difference (*P* < .05; Fig. 3).

### 3.4. Correlation between T2 values of different regions of knee cartilage and MRI osteoarthritis knee scores in the case group

There was no correlation between the T2 values of lateral femur and MRI osteoarthritis knee scores in the case group (*P* > .05), whereas there was a positive correlation between the T2 values of medial femur, lateral tibia, medial tibia, and patellar region and MRI osteoarthritis knee scores in the case group (*P* < .05; Table 2).The absence of a correlation in the lateral femur may be related to its distinct biomechanical environment. In typical KOA patterns, the medial compartment bears greater mechanical loading, while the lateral femur experiences lower loading forces and a different pattern of cartilage degeneration. These factors may reduce the sensitivity of lateral femur T2 values to changes captured by composite MRI scoring systems.

**Table 2:** Correlation between T2 values of different regions of knee cartilage and MRI osteoarthritis knee scores in the case group.

T2 value	MRI osteoarthritis knee scores	
	*r*	*P*
Lateral femur	0.014	.478
Medial femur	0.452	.002
Lateral tibia	0.623	.000
Medial tibia	0.775	.000
Patellar region	0.509	.001

MRI = magnetic resonance imaging.

### 3.5. Typical case

**Figure 4.**
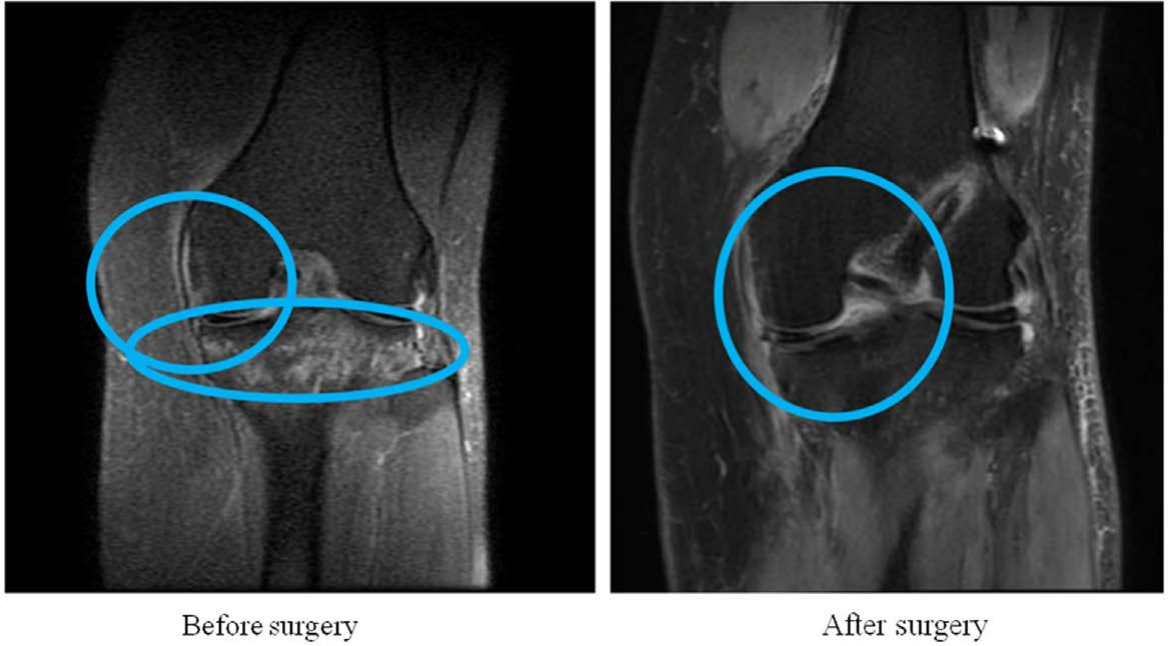
Case 1: Patient with preoperative medial cartilage damage to the distal femur and marrow damage to the proximal tibia.

**Figure 5.**
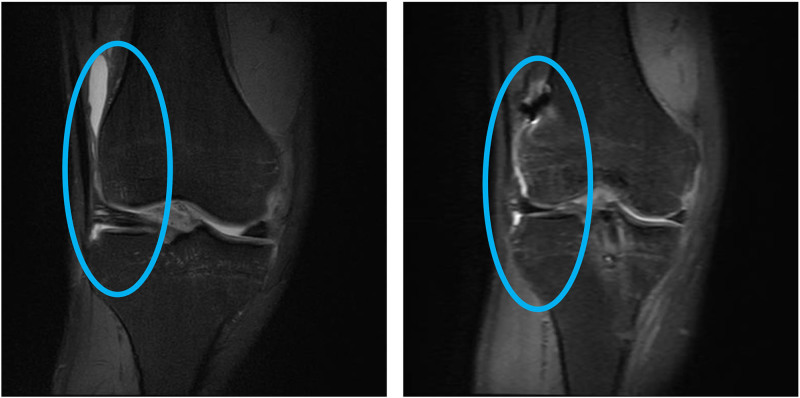
Case 2: Patient with preoperative anterior cruciate rupture and postoperative repair.

The imaging data and case analysis are shown in Figures 4 and 5.

## 4. Discussion

KOA is a prevalent degenerative joint disease, characterized by cartilage degeneration and loss, bone hyperplasia, and joint space narrowing.^[16–18,19]^ For patients with KOA, early detection combined with effective treatment can alleviate symptoms and slow disease progression, but reversing structural cartilage damage remains challenging due to its limited intrinsic repair capacity.^[20,21]^ The primary pathological feature of KOA is the degradation and damage of articular cartilage. Due to its lack of nerve and vascular supply, the cartilage has a limited capacity for self-healing or repair after degenerative changes. Cartilage damage not only causes joint pain but also exacerbates the destruction of joint structures and results in functional impairment.^[22]^

Currently, most conventional treatments such as medication and physical therapy can only provide short-term relief from patients’ pain symptoms, without fundamentally reversing or restoring the normal structure and function of cartilage. The prognosis for cartilage damage in KOA is influenced by various factors, but with early diagnosis, effective treatment, and a healthy lifestyle, patients’ quality of life can be significantly improved.^[23]^ The prognosis of KOA is affected by several factors, including the patient’s age, severity of the condition, choice of treatment, and compliance.^[24]^ While conventional treatments may alleviate symptoms to some extent, they struggle to reverse disease progression. Novel therapeutic approaches offer new options for KOA patients and have demonstrated promising outcomes in clinical trials, both in treatment efficacy and prognosis improvement.^[25]^

MRI, as a sensitive and noninvasive diagnostic tool, plays a crucial role in diagnosing KOA and assessing cartilage damage. MRI provides a clear depiction of the anatomical structures of the knee, including the articular cartilage, meniscus, ligaments, synovium, and joint cavity, offering unique advantages in evaluating cartilage damages, joint effusion, and osteophyte formation.^[22]^ MRI not only detects early cartilage lesions but also quantifies the severity of the lesions, providing critical support for clinical diagnosis and treatment.^[22]^ On MRI images, cartilage damage in KOA manifests as changes in cartilage signal, reduced cartilage thickness, and alterations in the subchondral bone. The T2 mapping sequence, as a specialized MRI technique, can reflect changes in water molecules, collagen fibers, and tissue anisotropy within cartilage, making it particularly sensitive to cartilage damage.^[26]^ The results of this study showed that preoperative T2 values in all examined regions were significantly higher in KOA patients than in controls, with postoperative values approaching control levels in most regions.

Compared with X-ray and CT, MRI is able to detect small lesions in cartilage earlier and accurately assess the degree of damage. In addition, MRI can also show changes in other structures within the joint, such as meniscus, ligaments, synovium, etc, providing comprehensive information for clinical diagnosis and treatment. The findings of this study demonstrate that MRI can noninvasively and clearly reveal morphological and structural changes in joint cartilage, with high sensitivity and specificity for cartilage damage. Compared to X-ray and CT, MRI can detect subtle cartilage lesions at an earlier stage and accurately assess the extent of damage. Additionally, MRI can display changes in other intra-articular structures, such as the meniscus, ligaments, and synovium, providing comprehensive information for clinical diagnosis and treatment.

Currently, various MRI grading systems are employed for the diagnosis and study of KOA, the more commonly used of which include the Boston Leeds Osteoarthritis Knee Score and the WORMS. These systems provide a quantitative assessment of lesions in different regions of the knee joint to comprehensively evaluate the severity of the disease. The WORMS scoring system is more comprehensive, which not only includes the elements of Boston Leeds Osteoarthritis Knee Score but also extends to the evaluation of structures such as ligaments and menisci.^[27]^ Our results support the application of such systems, particularly when combined with quantitative T2 mapping, to provide both morphological and compositional assessment and to monitor prognosis.

At the same time, MRI osteoarthritis knee score can also provide researchers with standardized methods of data collection and analysis and promote in-depth research in related fields.^[28]^ The application of MRI scoring system in cartilage damage in KOA has significant advantages, which can accurately and reliably assess the extent of cartilage damage and other changes in joint structure. With the continuous advancement of technology and in-depth promotion of its application, MRI osteoarthritis knee score is expected to become an important auxiliary approach for the diagnosis and treatment of KOA.^[29,30]^

Limitations of our study include the absence of an a priori sample size calculation (although retrospective power analysis confirmed adequate power), the need to adjust for multiple comparisons (Bonferroni correction applied in this revision), and the short postoperative follow-up (12 weeks), which limits long-term prognostic assessment. Future research with larger cohorts, longer follow-up, and full joint assessment using advanced 3D mapping could strengthen these findings.

In conclusion, this study demonstrates that combining T2 mapping with MRI osteoarthritis knee scoring yields complementary insights into cartilage damage and prognosis in KOA. The compartment-specific findings for the lateral femur and the clinical significance of residual postoperative abnormalities underscore the importance of region-focused imaging assessment and long-term monitoring.

## Author contributions

**Conceptualization:** Jinbao Li, Xiuwei Zheng.

**Data curation:** Jinbao Li, Xiuwei Zheng.

**Formal analysis:** Jinbao Li, Xiuwei Zheng.

**Investigation:** Jinbao Li.

**Methodology:** Jinbao Li, Xiuwei Zheng.

**Project administration:** Xiuwei Zheng.

**Validation:** Jinbao Li.

**Writing – original draft:** Jinbao Li, Xiuwei Zheng.

**Writing – review & editing:** Jinbao Li, Xiuwei Zheng.

## References

[1] Du X Liu ZY Tao XX. Research progress on the pathogenesis of knee osteoarthritis. Orthop Surg. 2023;15:2213–24. 37435789 10.1111/os.13809 PMC10475681

[2] Martín Noguerol T Raya JG Wessell DE Vilanova JC Rossi I Luna A. Functional MRI for evaluation of hyaline cartilage extracelullar matrix, a physiopathological-based approach. Br J Radiol. 2019;92:20190443. 31433668 10.1259/bjr.20190443 PMC6849690

[3] Fowkes MM Das Neves Borges P Cacho-Nerin F Brennan PE Vincent TL Lim NH. Imaging articular cartilage in osteoarthritis using targeted peptide radiocontrast agents. PLoS One. 2022;17:e0268223. 35536857 10.1371/journal.pone.0268223 PMC9089912

[4] Guo X Xi L Yu M. Regeneration of articular cartilage defects: therapeutic strategies and perspectives. J Tissue Eng. 2023;14:20417314231164765. 37025158 10.1177/20417314231164765 PMC10071204

[5] Yao Q Wu X Tao C. Osteoarthritis: pathogenic signaling pathways and therapeutic targets. Signal Transduct Target Ther. 2023;8:56. 36737426 10.1038/s41392-023-01330-w PMC9898571

[6] Herrera D Almhdie-Imjabbar A Toumi H Lespessailles E. Magnetic resonance imaging-based biomarkers for knee osteoarthritis outcomes: a narrative review of prediction but not association studies. Eur J Radiol. 2024;181:111731. 39276401 10.1016/j.ejrad.2024.111731

[7] Delpachitra SN Dimitroulis G. Osteoarthritis of the temporomandibular joint: a review of aetiology and pathogenesis. Br J Oral Maxillofac Surg. 2022;60:387–96. 35307273 10.1016/j.bjoms.2021.06.017

[8] Roemer FW Guermazi A Demehri S Wirth W Kijowski R. Imaging in osteoarthritis. Osteoarthritis Cartilage. 2022;30:913–34. 34560261 10.1016/j.joca.2021.04.018

[9] Zhao H Li H Liang S Wang X Yang F. T2 mapping for knee cartilage degeneration in young patients with mild symptoms. BMC Med Imaging. 2022;22:72. 35436880 10.1186/s12880-022-00799-1 PMC9017029

[10] Martel-Pelletier J Tardif G Paiement P Pelletier JP. Common biochemical and magnetic resonance imaging biomarkers of early knee osteoarthritis and of exercise/training in athletes: a narrative review. Diagnostics (Basel). 2021;11:1488. 34441422 10.3390/diagnostics11081488 PMC8391340

[11] Hayashi D Roemer FW Link T. Latest advancements in imaging techniques in OA. Ther Adv Musculoskelet Dis. 2022;14:1759720X221146621. 10.1177/1759720X221146621 PMC9806406 36601087

[12] Harlianto N Hirvasniemi J Poot D. T2 mapping of the articular cartilage as a biomarker for oa in a population based cohort: the Rotterdam study. Osteoarthritis Imaging. 2022;2:100023. 10.1016/j.joca.2025.09.009 40975370

[13] Tong L Yu H Huang X. Current understanding of osteoarthritis pathogenesis and relevant new approaches. Bone Res. 2022;10:60. 36127328 10.1038/s41413-022-00226-9 PMC9489702

[14] Nagy EE Nagy-Finna C Popoviciu H Kovács B. Soluble biomarkers of osteoporosis and osteoarthritis, from pathway mapping to clinical trials: an update. Clin Interv Aging. 2020;15:501–18. 32308378 10.2147/CIA.S242288 PMC7152733

[15] Aubourg G Rice SJ Bruce-Wootton P Loughlin J. Genetics of osteoarthritis. Osteoarthritis Cartilage. 2022;30:636–49. 33722698 10.1016/j.joca.2021.03.002 PMC9067452

[16] Li S Jiang X Wang J. Clinical efficacy of 2-needle joint lavage for osteoarthritis-related knee pain and predictors of response based on knee MRI osteoarthritis knee score: a medical records review study. J Clin Rheumatol. 2023;29:396–401. 37779229 10.1097/RHU.0000000000002029

[17] Zhu X Xu H Wang L. Impact of lateral meniscus injury detected by preoperative magnetic resonance imaging on midterm results after unicompartmental knee arthroplasty. Knee. 2023;44:227–35. 37677873 10.1016/j.knee.2023.08.014

[18] Afsahi AM Sedaghat S Moazamian D. Articular cartilage assessment using ultrashort echo time MRI: a review. Front Endocrinol (Lausanne). 2022;13:892961. 35692400 10.3389/fendo.2022.892961 PMC9178905

[19] Peuna A Thevenot J Saarakkala S Nieminen MT Lammentausta E. Machine learning classification on texture analyzed T2 maps of osteoarthritic cartilage: oulu knee osteoarthritis study. Osteoarthritis Cartilage. 2021;29:859–69. 33631317 10.1016/j.joca.2021.02.561

[20] Shibata K Wakasa M Saito A. Hyperechoic and low morphological changes in the prefemoral fat pad in individuals with knee osteoarthritis based on ultrasonographic findings. J Med Ultrasound. 2021;29:105–10. 34377641 10.4103/JMU.JMU_85_20 PMC8330681

[21] Panos JA Webster KE Hewett TE. Anterior cruciate ligament grafts display differential maturation patterns on magnetic resonance imaging following reconstruction: a systematic review. Knee Surg Sports Traumatol Arthrosc. 2020;28:2124–38. 31520146 10.1007/s00167-019-05685-y PMC7067650

[22] Ota S Sasaki E Sasaki S. Relationship between abnormalities detected by magnetic resonance imaging and knee symptoms in early knee osteoarthritis. Sci Rep. 2021;11:15179. 34312418 10.1038/s41598-021-94382-3 PMC8313522

[23] Coaccioli S Sarzi-Puttini P Zis P Rinonapoli G Varrassi G. Osteoarthritis: new insight on its pathophysiology. J Clin Med. 2022;11:6013. 36294334 10.3390/jcm11206013 PMC9604603

[24] Weber AE Bolia IK Trasolini NA. Biological strategies for osteoarthritis: from early diagnosis to treatment. Int Orthop. 2021;45:335–44. 33078204 10.1007/s00264-020-04838-w

[25] Sabha M Hochberg MC. Non-surgical management of hip and knee osteoarthritis; comparison of ACR/AF and OARSI 2019 and VA/DoD 2020 guidelines. Osteoarthr Cartil Open. 2022;4:100232. 36474466 10.1016/j.ocarto.2021.100232 PMC9718349

[26] D’Agostino V Sorriento A Cafarelli A. Ultrasound imaging in knee osteoarthritis: current role, recent advancements, and future perspectives. J Clin Med. 2024;13:4930. 39201072 10.3390/jcm13164930 PMC11355885

[27] Nevalainen MT Uusimaa AP Saarakkala S. The ultrasound assessment of osteoarthritis: the current status. Skeletal Radiol. 2023;52:2271–82. 37060461 10.1007/s00256-023-04342-3 PMC10509065

[28] Bodden J Ok AH Joseph GB. Joint-adjacent adipose tissue by MRI is associated with prevalence and progression of knee degenerative changes: data from the osteoarthritis initiative. J Magn Reson Imaging. 2021;54:155–65. 33644919 10.1002/jmri.27574 PMC8211379

[29] Newman S Ahmed H Rehmatullah N. Radiographic vs. MRI vs. arthroscopic assessment and grading of knee osteoarthritis - are we using appropriate imaging? J Exp Orthop. 2022;9:2. 34978625 10.1186/s40634-021-00442-y PMC8724325

[30] Sharma L. Osteoarthritis of the knee. N Engl J Med. 2021;384:51–9. 33406330 10.1056/NEJMcp1903768

